# Structure and Bioactivity Screening of a Low Molecular Weight Ulvan from the Green Alga *Ulothrix flacca*

**DOI:** 10.3390/md16080281

**Published:** 2018-08-15

**Authors:** Peipei Li, Songsong Wen, Kunlai Sun, Yuqin Zhao, Yin Chen

**Affiliations:** 1 Marine School, Ningbo University, 315000 Ningbo, China; Lipeipei1985@163.com; 2 Zhejiang Mariculture Research Institute, Zhoushan 316000, China; 3 Shandong Institute of Food and Drug Control, Jinan 250000, China; songsongwen2010@126.com; 4 College of Food and Pharmacy, Zhejiang Ocean University, Zhoushan 316000, China; sunqinlai@126.com (K.S.); Zhaoy@zjou.edu.cn (Y.Z.)

**Keywords:** green algae, ulvan, structure, anticoagulant activity, immunoregulation

## Abstract

A water-soluble low molecular–weight polysaccharide named UP2-1 was isolated and purified from the marine green algae *Ulothrix* *flacca* using ion-exchange and size-exclusion chromatography. Composition and characteristics analyses showed that UP2-1 was a sulfated glucuronorhamnan consisting of rhamnose and glucuronic acid in a ratio of 2:1 with 21% sulfate content and a molecular weight of 5.0 kDa. Structural properties were determined using desulfation and methylation analyses combined with infrared spectrum (IR), gas chromatography-mass spectrometer (GC-MS) and nuclear magnetic resonance (NMR). The results showed that UP2-1 was a type of ulvan composed of alternate 4-linked-α-L-rhamnose residues (→4)-α-L-Rha(1→) and 4-linked-β-D-glucouronoc acid residues. The sulfate groups were mainly present in the *O*-3 position of →4)-α-L-Rha(1→. Most (70%) of the rhamnose was sulfated. UP2-1 also had a small amount of →4)-α-L-Rha(1→ branch at the *O*-2 position of the →4)-α-L-Rha(1→. UP2-1 exhibited significant anticoagulant and immunomodulating activity in vitro. This study demonstrated that the green algae *Ulothrix flacca*, which is used as a food and traditional marine herb in China, could also be considered as a source of bioactive ulvan.

## 1. Introduction 

Polysaccharides from marine algae have special structural and functional properties that have attracted considerable attention for a range of applications [1,2]. Their broad pharmacological activities make them a rich and valuable source for biomaterial and drug development. Red and brown algae are sources of alginate, carrageenan, agar, and fucoidan [3,4,5]. Fucoidan is one of the best-studied marine polysaccharides and is the major sulfated polysaccharide of brown algae [6]. It has various antagonist effects against angiogenesis, coagulation, inflammation, metastasis, viruses, tumors, and oxidation and also has immunoregulatory functions [7,8].

Green algae are the most abundant marine algae [9]. They also contain sulfated polysaccharides with special structures and bioactivities, such as ulvan, that have been little investigated [10,11]. Ulvan is a sulfated polysaccharide obtained from several Ulvales species and is composed mainly of rhamnose, glucuronic acid, and to a lesser extent iduronic acid and xylose [12]. Other sulfated polysaccharides found in different green algae species include rhamnan, arabinan, and galactan. A sulfated polysaccharide named PML, isolated from *Monostroma latissimum* (Kuetzing) Wittrock, is high in rhamnose as (1→3)-linked-L-rhamnopyranose [13]. Another sulfated polysaccharide named FEP, isolated from *Enteromorpha clathrata* (Roth) Grev., is a sulfated arabinan with a sulfate ester content of 31.0% [14]. FEP is comprised mainly of (1→4)-linked α-L-arabinopyranose residues with sulfate groups at the *O*-3 position. CP2-1 is also a sulfated polysaccharide and is isolated from another species of green algae, *Codium divaricatum* Holmes*.* It is a highly sulfated galactan with pyruvic acid ketal substitutions [15]. Most sulfated polysaccharides possess noticeable anticoagulant activity and can prolong the activated partial thromboplastin time and thrombin time effectively. Thus, green algae are another source of bioactive polysaccharides.

*Ulothrix flacca* (Dillwyn) Thuret, a green macroalgae, grows along most coastlines of China and widely across the world, including Europe and the Americas. In China, it is used as a food and as a traditional marine herb for resolving hard lumps and edema. However, the structures and biological activities of compounds from this alga have not been well studied. As a renewable resource, its potential values and applications need to be explored.

## 2. Results and Discussion

### 2.1. Polysaccharide Purification and Purity 

**Figure 1:**
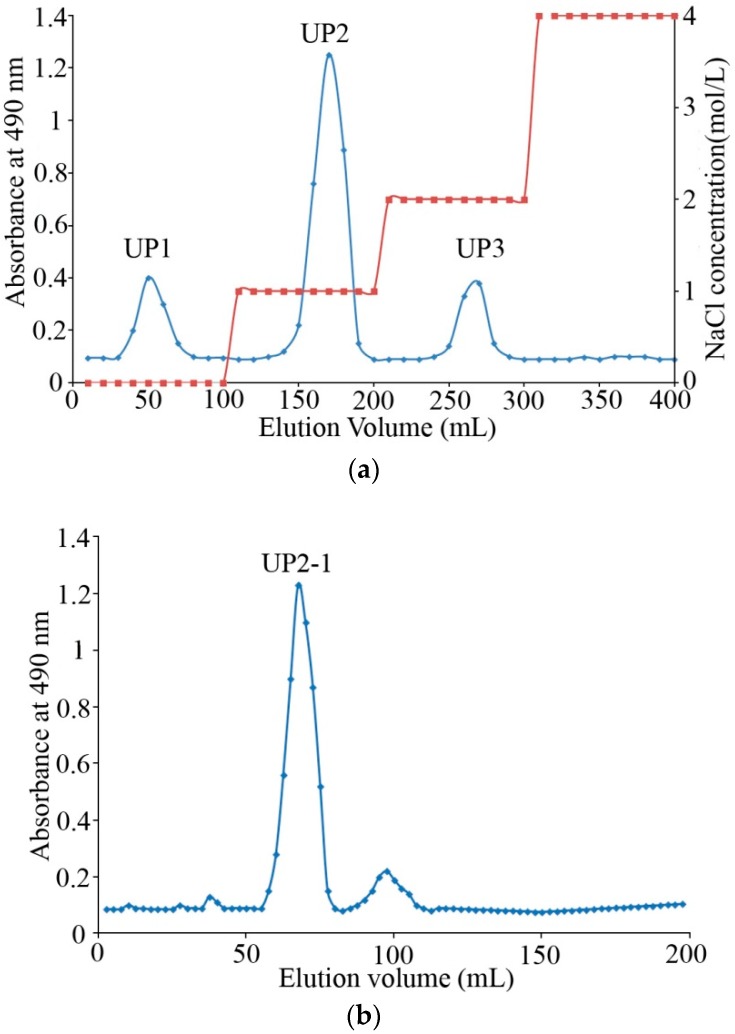

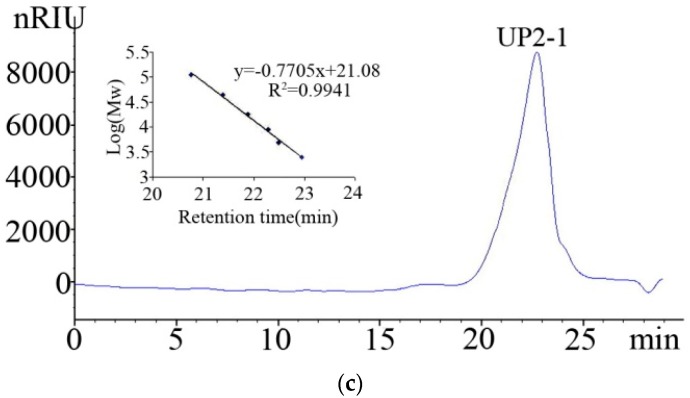
Isolation and high performance gel permeation chromatography (HPGPC) chromatogram of the polysaccharides obtained from the marine algae, *U. flacca*. (**a**) Crude polysaccharides eluted from the Q Sepharose Fast Flow column, UP1-3 means three eluted polysaccharide fractions from the column in order; (**b**) purification of UP2 on a Superdex75 column, UP2-1 means the purified UP2; (**c**) High performance gel permeation chromatography (HPGPC) chromatograms of UP2-1 on a TSKgel G3000PWxl column (7.8 mm × 30.0 cm) and standard curve of molecular weights.

The crude polysaccharides extracted from *U. flacca* were approximately 25% (*w/w*) of dry algae powder. Q Sepharose Fast Flow column chromatography was used to separate polysaccharides with 0, 1.0, and 2.0 mol/L NaCl (Figure 1a). Afterward, 1.0 mol/L NaCl eluted fraction UP2, as the major component with the best solubility and crude anticoagulant activity, was chosen. UP2 was concentrated, further purified on a Superdex 75 column, and collected according to the phenol-H_2_SO_4_ detection method and named UP2-1 (Figure 1b).

UP2-1 showed as a single sharp peak in the HPGPC chromatogram, revealing that it was a relatively homogeneous polysaccharide (Figure 1c). Its molecular weight was estimated to be about 5 kDa according to the standard calibration curve. This was lower than other algae-derived polysaccharides, which are often in the range of 200–870 kDa [15]. UP2-1 is a natural low molecular weight polysaccharide in *U. flacca*. 

### 2.2. Composition Analysis

**Figure 2:**
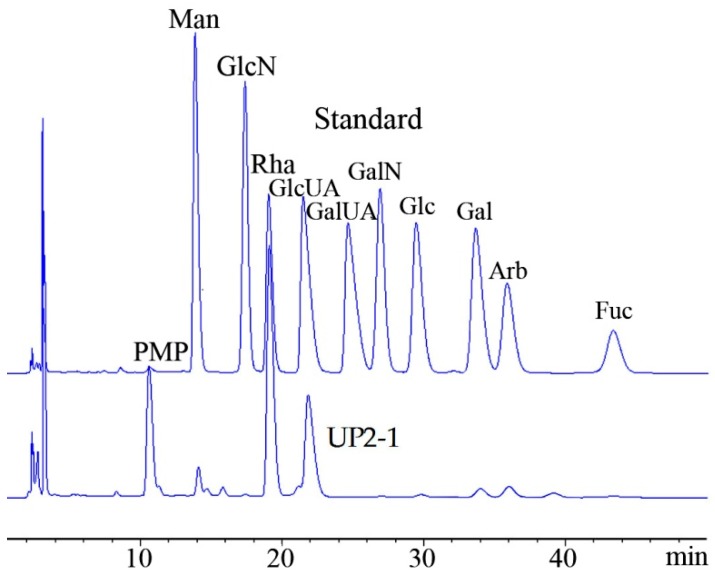
HPLC chromatogram of the 1-phenyl-3-methyl-5-pyrazolone pre-column derivative, UP2-1, for monosaccharide composition with standards (Man: d-mannose, GlcN: d-glucosamine, Rha: l-rhamnose, GlcUA: d-glucouronic acid, GalUA: d-galactouronic acid, Glc: d-glucose, Gal: d-galactose, Arb: d-arbinose, Fuc: l-fucose, PMP: 1-Phenyl-3-methyl-5-pyrazalone, derivative reagent).

The study of chemical characteristics revealed that UP2-1 contained sulfate ester and uronic acid at 21% and 20%, respectively. No protein was detected. Monosaccharide composition analysis by high performance liquid chromatography (HPLC) showed that UP2-1 consisted mainly of rhamnose and glucuronic acid at a ratio of 2.1:1.0 (Figure 2). The results showed that UP2-1 was a sulfated acidic polysaccharide containing abundant rhamnose. The uronic acid in UP2 was glucuronic acid. These data indicated that the polysaccharide, UP2-1, could be a type of ulvan.

### 2.3. Linkage Analysis

As a sulfated polysaccharide, besides linkage styles, the sulfate group position is also important structural information related to bioactivity [16]. The classic method for obtaining structural information is methylation analysis of the sulfated polysaccharide and its desulfation sample. Fourier-transform infrared spectroscopy (FT-IR) is a convenient tool to evaluate methylation and desulfation processes.

**Figure 3:**
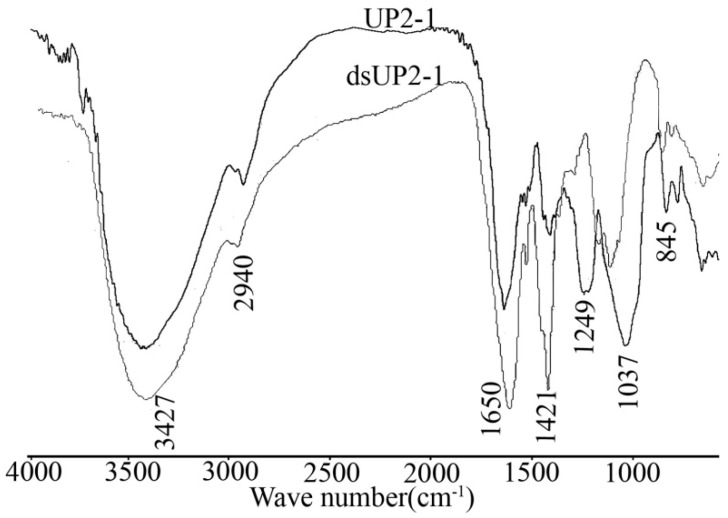
Infrared (IR) spectrum of UP2-1 and its desulfation product, dsUP2-1.

The FT-IR spectrum of the sulfated polysaccharide, UP2-1, is shown in Figure 3. The broad and intense signal at 3247 cm^−1^ was characteristic of the stretching vibration bend of the O–H of sugar. The peaks at 2940 and 1421 cm^−1^ were attributed to the C–H stretching vibration and deformation vibration, respectively. The intense band at 1646 cm^−1^ corresponded to the carboxylic group within the glucuronic acid of the polysaccharide. The band at about 1051 cm^−1^ related to stretching vibrations of the C–O–C bond and glycoside bridge. The signal at 1249 cm^−1^ corresponded to S=O stretching vibrations, while the signals at 845 cm^−1^ were assigned to the C–O–S bending vibration of the sulfate ester in the axial position. These two peaks were also evidence for the existence of a sulfate group in UP2-1. Completion of the methylation reaction was confirmed by the disappearance of the O–H signal at 3247 cm^−1^ and the intense increase of CH_3_ at 2940 and 1421 cm^−1^ [15]. Desulfation was confirmed by the disappearance of the C–O–S and S=O bands at 1249 cm^−1^ and 845 cm^−1^, respectively (Figure 3).

The chromatography-mass spectrometer (GC-MS) results for partially *O*-methylated alditol acetates of UP2-1 (Table 1) showed that there were four significant linkages, all of which were identified positively using the standard database in partially methylated alditol acetates (PMAA). In UP2-1, Rha mainly had the linkages of (1→4)-linked-L-rhamnopyranose (→4)Rha*p*(1→), (1→3)-linked-L-rhamnopyranose (→3)Rha*p*(1→) and the branched linkage of (1→3,4)-linked-L-rhamnopyranose (→3,4)Rha*p*(1→) and (1→2.4)-linked-L-rhamnopyranose (→2,4)Rha*p*(1→) at a ratio of 1.0:1.1:7.8:1.2. →3,4)Rha*p*(1→ was the major linkage, accounting for 70% of the total linkage. Although UP2-1 contained about one-third of GlcA in a monosaccharide composition, it was difficult to get linkage information from GC-MS directly as methylated GlcA is challenging to gasify at normal conditions. After desulfation, the ratio of the four linkages changed greatly compared to the original composition. →4)Rha*p*(1→ increased from 9% to 67%, while →3,4)Rha*p*(1→decreased from 70% to 8%. In contrast, there were no obvious changes in →3)Rha*p*(1→ and →2,4)Rha*p*(1→ content before and after desulfation. From these results, it was presumed that UP2-1 had the linkages of →3)Rha*p*(1→, →4)Rha*p* (1→ and the branch of →2, 4)Rha*p*(1→. The sulfate group was substituted at *O*-3 position of →4)Rha*p*(1→.

### 2.4. Nuclear Magnetic Resonance (NMR) Analysis

**Figure 4:**
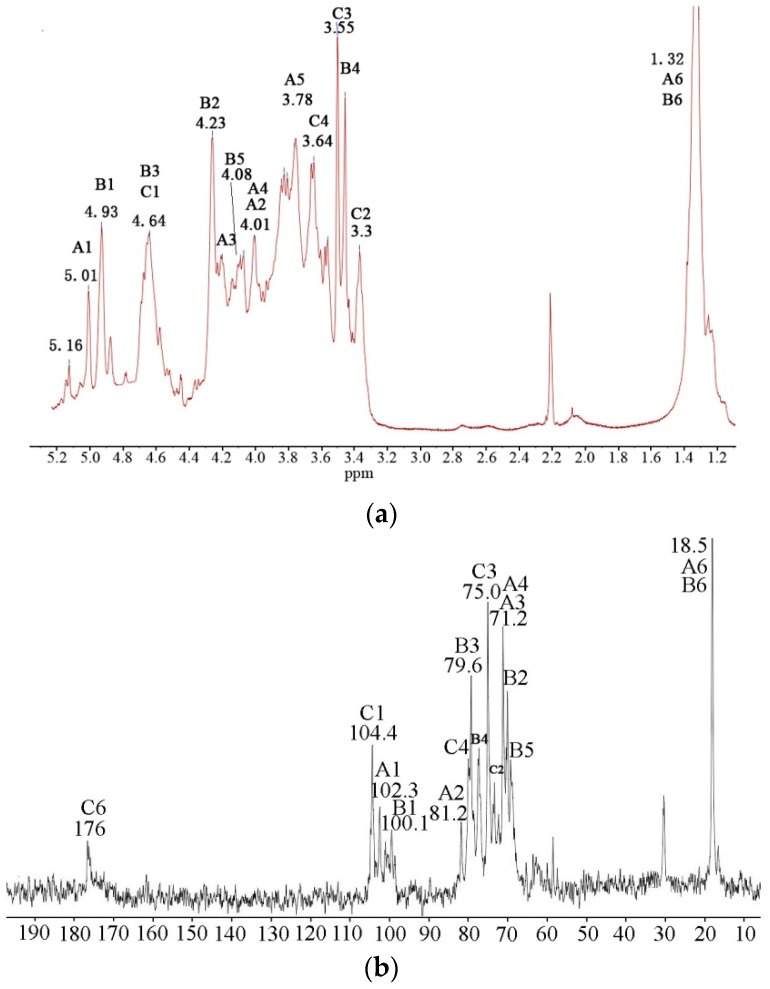

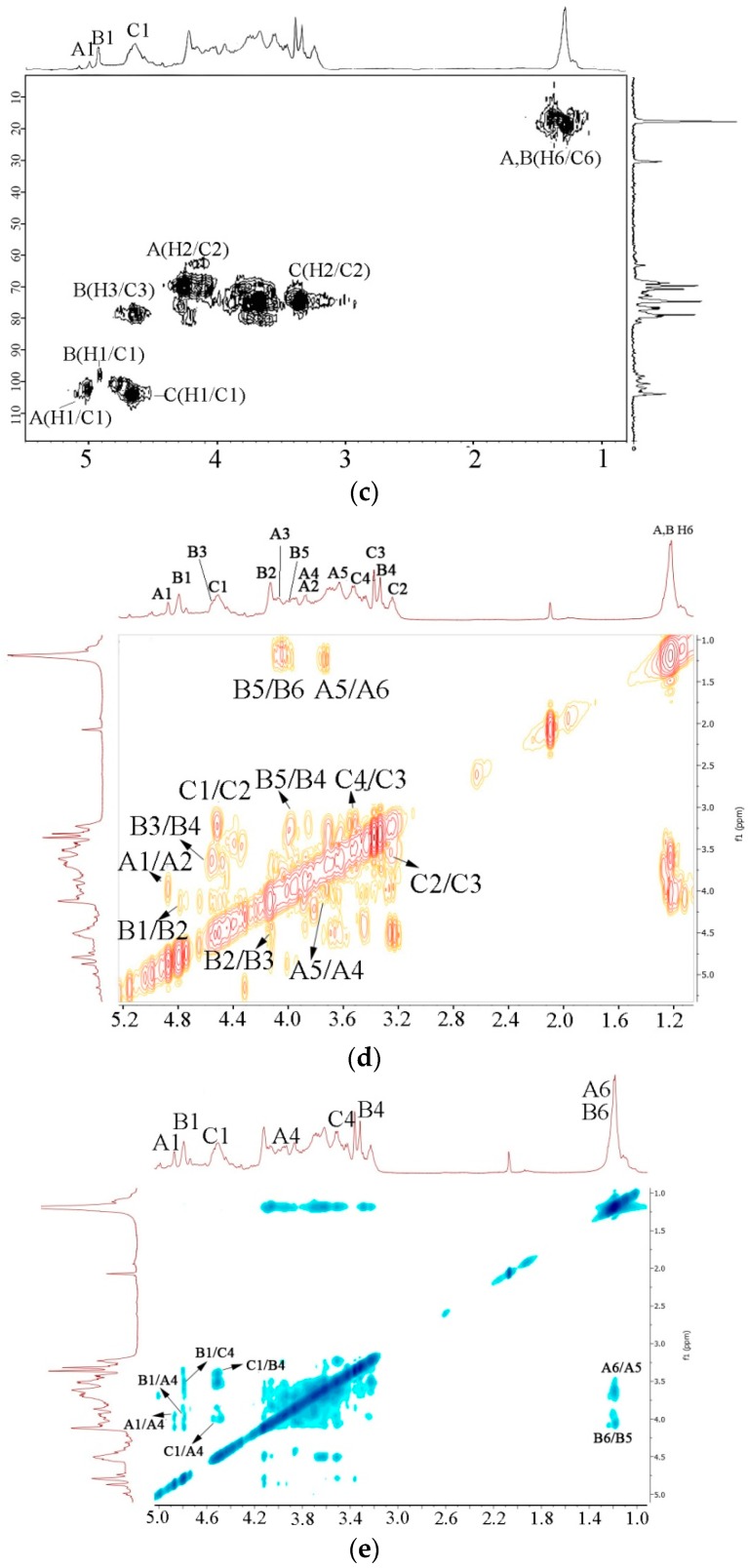
NMR spectra of UP2-1. Spectra were performed at 23 °C on a JEOL ECP 600 MHz spectrometer using acetone as the internal standard. (**a**) ^1^H NMR spectrum; (**b**) ^13^C NMR spectra; (**c**) ^1^H–^13^C HMQC spectrum; (**d**) ^1^H–^1^H COSY spectrum; (**e**) ^1^H–^1^H NOESY spectrum. A–C correspond to (1→4)-α-L-Rha*p*, (1→4)-α-L-Rha3S and (1→4)-β-D-GlcA, respectively. A1, B1 and C1 means the H1 or C1 signal of residues **A**, **B** and **C**, and so on.

To understand the linkage sequences, sugar configuration and linkages of GlcA in detail, NMR analysis was undertaken on UP2-1. In the ^1^H NMR spectrum of UP2-1 (Figure 4a), three major proton signals at 5.16, 5.01 and 4.93 of the anomeric region were assigned to the H1 signals of (1→2,4)-linked α-L-Rha*p*, (1→4)-linked α-L-Rha*p* and (1→4)-linked 3-sulfated-α-L-Rha*p,* respectively, after comparison with references [16]. The small signal near 4.93 ppm was assigned to be also (1→4)-linked 3-sulfated-α-L-Rha*p* at different chemical environment. The strong signal at 4.64 ppm was characteristic of β-D-configured GlcA, (1→4)-linked β-D-GlcA residues. The signal at 1.32 ppm was typical for the CH_3_ group of Rha. Based on the ^1^H-^13^C heteronuclear multiple quantum coherence (HMQC) spectrum (Figure 4c), anomeric signals in the ^13^C NMR spectrum of UP2-1 (Figure 4b) were also assigned. The signal at 104.4 ppm, which was related to the proton signal at 4.64 ppm in HMQC spectrum, was attributed to (1→4)-linked β-D-GlcA residues. The signal at 102.1 ppm was deduced to be C1 of (1→4)-linked α-L-Rha*p*, whereas 100.1 ppm was the C1 signal of (1→4)-linked 3-sulfate-α-L-Rha*p*. The signals at 18.5 and 176 ppm were attributed to the C6 of Rha and GlcA, respectively.

The combination of HMQC and ^1^H–^1^H correlation spectroscopy (COSY) spectrum (Figure 4d) of UP2-1 allowed assignment of the major signals of the sugar spin systems (Table 2). From the spectrum, we obtained the typical characteristic of (1→4)-linked α-L-Rha*p*, H4 and C4, which changed to low displacement at 3.96 and 81.4 ppm, respectively. Compared with the data from (1→4)-linked α-L-Rha*p*, the chemical shifts of (1→4)-linked 3-sulfate-α-L-Rha*p* changed greatly. After 3-*O* sulfation, H3 changed from about 4.1 ppm to 4.6 ppm and overlapped with the anomeric signal of GlcA, while C3 changed to 79.5 ppm. 3-*O* sulfation also affected the chemical shift of H2, which changed to low field at 4.2 ppm. Although the signals of (1→4)-linked β-D-GlcA were not completely assigned, it was determined that (1→4)-linked β-D-GlcA in UP2-1 had no sulfation modification. This was determined because the spin system chemical shifts did not change compared with the standard NMR data of (1→4)-linked β-D-GlcA [17].

Nuclear overhauser effect spectroscopy (NOESY) spectrum (Figure 4e) of UP2-1 had a linkage sequence of different glycosidic residues. The strong cross peak H-1/H-4 of **A** confirmed the main (1→4)-linked α-L-Rha*p*. The cross signal of the H-1 of **A** and H-4 of **B** suggested that the (1→4)-linked α-L-Rha*p* was linked to the C-4 of the (1→4)-linked 3-sulfate-α-L-Rha*p* residues. The signal of H-1 of **C** and H-4 of **A** and **B** indicated that (1→4)-linked β-D-GlcA was linked to C-4 of the (1→4)-linked 3-sulfate-α-L-Rha*p* or (1→4)-linked α-L-Rha*p*.

**Figure 5:**
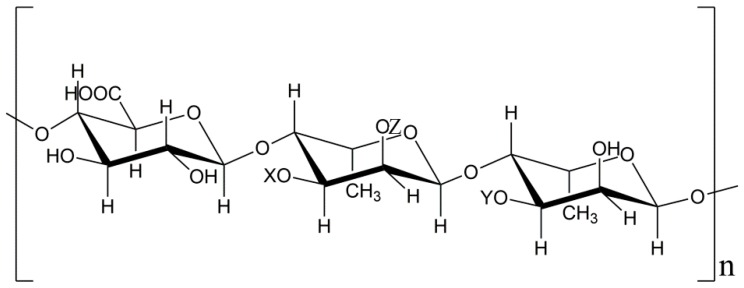
Proposed structure of UP2-1. (X represented SO_3_^−^ or branch position, Y mainly represented H, Z could be H or branch position, X:Y = 7:3).

Complete assignment of NMR spectra of UP2-1 was difficult because of overlapping signals. Thus, further elucidation of the precise structures was limited. From the NMR, we could not recognize the signals of →3)Rha*p*(1→ or →2,4)Rha*p*(1→. However, with combined results UP2-1 was deduced to consist of the main chain of (1→4)-linked α-L-Rha*p* and (1→4)-linked β-D-GlcA in a 2:1 ratio. Most (75%) of (1→4)-linked α-L-Rha*p* had the sulfation modification at the 3-*O* position, while 10% were branched at the 2-*O* position. The predicted structure of UP2-1 is shown in Figure 5.

Ulvan is a type of sulfated polysaccharide, composed mainly of Rha and GlcA in different structures that is abundant in the green algae of *Ulva*, *Enteromorpha*, and *Monostroma* [18]. *Monostroma* polysaccharides are composed of (1→3)-linked α-L-Rha*p* and (1→2)-linked α-L-Rha*p* with sulfation groups at 3-*O* or 2-*O* positions, with much lower GlcA content. UP2-1 is similar to the polysaccharides from *Ulva* and *Enteromorpha*, which are composed of repeating disaccharides unit of →4)-β-D-GlcA-(1→4)-α-L-Rha3-sulfate and some other monosaccharides such as xylose, arabinose, and glucose. Compared with the ulvan from *Enteromorpha*, UP2-1 had higher GlcA and sulfate content but a smaller molecular weight.

### 2.5. Bioactivity Screening

UP2-1 was subjected to bioactivity screening. Anticoagulant activities of the sulfated polysaccharide were evaluated by activated partial thromboplastin time (APTT), thrombin time (TT), and prothrombin time (PT) assays with heparin for comparison. As shown in Table 3, UP2-1 prolonged APTT and TT effectively. The clotting time for UP2-1 20 μg/mL was more than 200 s for APTT and 120 s for TT, while PT was not prolonged. Coagulation includes intrinsic and extrinsic pathways, with APTT relating to the former and PT to the latter. TT relates to thrombin activity or fibrin polymerization. For UP2-1, the lack of PT prolongation activity demonstrated no inhibition of the extrinsic coagulation pathway. Thus, it was presumed that UP2-1 acted on the intrinsic coagulation pathway, and via effects on thrombin activity or conversion of fibrinogen to fibrin [18]. APTT and TT activities were increased rapidly by heparin. Compared with heparin, UP2-1 had mild anticoagulant activities similar to those of low molecular weight heparins (LMWHs) [19]. LMWHs are derived from heparin by polydisperse depolymerization and have molecular weights under 6 kDa. They are used widely as anticoagulants and have half the activity of heparin but less bleeding risk [20]. UP2-1 could be investigated as an alternative to LMWHs.

**Figure 6:**
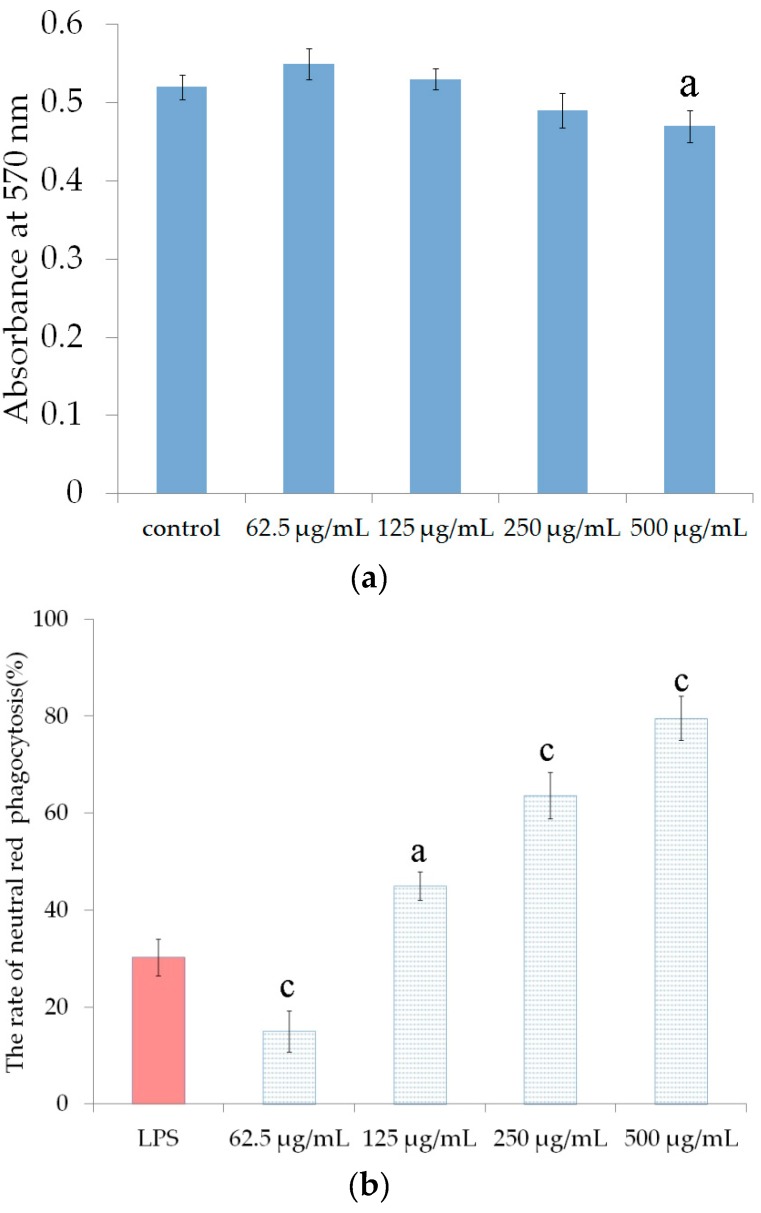
Immunoregulatory activity of UP2-1. (**a**) Effects of UP2-1 on RAW264.7 cell proliferation; (**b**) Phagocytotic activity of RAW264.7 cells stimulated by UP2-1. Each value is expressed as mean ± SD (n = 3). LPS means lipopolysaccharide. Significant differences with model control group were designated as a: *p* < 0.05, b: *p* < 0.01 and c: *p* < 0.001.

Enhanced immunity and immune response is a potential method for inhibiting tumor growth without significant side effects for the host [21]. Therefore, it is important to explore new non-toxic immunomodulatory substances that can be used as adjuvant chemotherapy. Many marine polysaccharides have immunomodulatory activity, especially those of low molecular weight [22,23]. To investigate the effects of UP2-1 on RAW264.7 (mouse leukemia cells of monocyte macrophage) cell growth, cells were treated with polysaccharide 0-500 μg/mL for 24 h before assessing viability with the MTT (3-(4,5)-dimethylthiahiazo (-z-y1)-3,5-di- phenytetrazoliumromide) assay. UP2-1, in a concentration up to 500 μg/mL, was not cytotoxic to RAW264.7 cells (Figure 6a), and did not significantly affect cellular proliferation. The effect of UP2-1 on the pinocytic activity of RAW264.7 cells was examined by neutral red uptake. UP2-1 significantly and dose-dependently enhanced the pinocytic activity of RAW264.7 cells (Figure 6b). The increased phagocytosis rate was up to 78.5% at 500 μg/mL. These data indicated that UP2-1 could enhance macrophage phagocytosis activity.

UP2-1 exhibited good bioactivities, which might partially relate to its low molecular weight. As the molecular weight of sulfated polysaccharides increases, the solubility, absorptivity, and bioavailability decreases, potentially limiting application. Many methods have been developed to prepare low molecular weight polysaccharides with similar or better bioactivities [23,24]. As a natural low molecular weight sulfated polysaccharide, UP2-1 is worthy of further study and utilization.

## 3. Materials and Methods 

*Ulothrix flacca* (Dillwyn) Thuret was collected from the eastern coastal area of Zhoushan, China, east longitude 123° north latitude 30° at the end of May. After collection, the seaweed was cleaned with fresh water to remove sand and other absorbed impurities. We gently squeeze out the extra water and dried the sample in the sun for a day. Then, they were placed in an oven at 40 °C to further dry. When the algal body was dry and crispy, it was milled to a uniform powder.

### 3.1. Polysaccharide Isolation and Purification 

Polysaccharide extraction and purification was performed as described previously [25]. Briefly, dry algae powder (200 g) was immersed in distilled water (2 L) at 80 °C for 4 h under reflux. After the aqueous extract was filtered, the residue was extracted repetitively. The filtrate was combined and concentrated to about 1/10 of the original volume in a rotary evaporator under reduce pressure. Polysaccharides were recovered using 80% ethanol precipitation. After centrifugation (6000× *g* for 15 min), the precipitate was dissolved in distilled water. The resultant precipitate was removed by centrifugation, and the supernatant collected. The final solution was dialyzed (molecular weight cut-off 3 kDa) with distilled water for 48 h, and the white powder of crude polysaccharide obtained by freeze-drying with a yield of 25 % (*w*/*w*, in dry mass). 

The crude polysaccharide (100 mg) was dissolved in distilled water (2 mL) and purified by a Q Sepharose Fast Flow column (300 × 30 mm) (GE Healthcare, Stockholm, Sweden) coupled to an AKTA FPLC system (Purifier100, GE Healthcare, Stockholm, Sweden) eluted with a linear gradient of 0–4 mol/L NaCl. Fractions were collected and analyzed for carbohydrate content by the phenol–sulphuric acid method. Individual tubes of the major carbohydrate fraction were pooled, dialyzed exhaustively (molecular weight cut-off 3 kDa), and further purified on a Superdex 75 column (100 × 2 cm) (GE Healthcare, Stockholm, Sweden) eluted with 0.2 mol/L NH_4_HCO_3_ at a flow rate of 0.3 mL/min. The major polysaccharide fractions were pooled, freeze-dried, and named UP2-1. Purity and molecular weight were determined by high performance gel permeation chromatography (HPGPC) as described elsewhere [26].

### 3.2. Composition Analysis

UP2-1 (5 mg) hydrolysis was performed with 2 mol/L trifluoroacetic acid (1 mL) at 105 °C for 6 h. After trifluoroacetic acid removal by repeated co-evaporation with methanol, the hydrolysate was dried under reduced pressure. The monosaccharide composition was then analyzed by HPLC through pre-column derivatization with 1-phenyl-3-methyl-5-pyrazolone using an Agilent HPLC system fitted with Agilent XDB-C18 (4.6 × 250 mm) and Agilent XDB-UV detector. The sulfate content of the polysaccharide was determined by high-performance anion-exchange chromatography with pulsed amperometric detection (HPAEC-PAD) on a CIC-100 ion chromatograph (Thermo Fisher Scientific, Walthsam, MA, USA) coupled with an SH-AC-1 anion exchange column (4.6 mm × 250 mm, 13 µm) (ShengHan Chromatograph Technology, Qingdao, China) and a conductivity detector [26].

### 3.3. Linkage Analysis

Methylation analysis was performed using the method of Hakomori [27] with some modification. Each sample in dimethyl sulfoxide was methylated using NaH and iodomethane, with the completeness of the methylation confirmed by IR spectroscopy. After hydrolysis with 2 mol/L trifluoroacetic acid at 105 °C for 6 h, the methylated sugar residues were converted to partially methylated alditol acetates by reduction with NaBH_4_, followed by acetylation with acetic anhydride. The derived sugar residues were extracted into dichloromethane, evaporated to dryness, dissolved again in 100 μL dichloromethane, and then analyzed by gas chromatography–mass spectrometry (GC–MS) (Thermo Fisher Scientific, Walthsam, MA, USA). The products were analyzed by GC–MS on DB 225 using a temperature gradient: first at 100–220 °C at a rate of 5 °C/min and then keeping at 220 °C for 15 min. The chromatogram peaks were identified from their retention times. GC–MS was performed on an HP6890II instrument [26].

To determine the position of the sulfate groups on the sugar rings of UP2-1, desulfation of the sulfated polysaccharide was performed according to the reference [14,28]. Briefly, UP2-1 (30 mg) was dissolved in water and passed through a 732 cation-exchange resin column (H^+^ form), which was eluted with distilled water. The combined effluent was adjusted to pH 9.0 with pyridine and then lyophilized to give a white powdered pyridinium salt. The product was dissolved in 10 mL of dimethylsulfoxide containing 10% (*v*/*v*) anhydrous methanol and 1% pyridine, and then the solution was shaken at 100 °C for 4 h. After the reaction was complete, the mixture was dialyzed against distilled water, freeze-dried, and named dsUP2-1.

DsUP2-1 also underwent methylation analysis. Through comparison of the methylation analysis results for UP2-1 and dsUP2-1, we obtained information about the linkage styles and sulfation position. The effect of methylation and desulfation was confirmed by IR.

### 3.4. NMR Spectroscopy

^1^H and ^13^C NMR spectra were recorded at 23 °C using a JEOL JNM-ECP 600 MHz spectrometer (JEOL, Tokyo, Japan). Herein, 50 mg polysaccharides were deuterium exchanged by successive freeze-drying steps in 99% D_2_O and then dissolved in 0.5 mL of 99.98% D_2_O. Chemical shifts were expressed in ppm using acetone as internal standard at 2.225 ppm for ^1^H and 31.07 ppm for ^13^C. 2D ^1^H-^1^H COSY, ^1^H-^13^C HMQC, and ^1^H-^13^C HMBC experiments were also carried out. 

### 3.5. Assay of Bioactivity Screening

In vitro anticoagulant and immunomodulatory activities were screened.

In vitro anticoagulant activity was researched through APTT, TT, and PT assay. For APTT assay, briefly, 90 μL of citrated normal human plasma and 10 μL of sample solution (0–50 μg/mL) were incubated at 37 °C for 60 s. Then, 100 μL of prewarmed APTT assay reagent (JianCheng Bioengineering, Nanjing, China) was added to react at 37 °C for 2 min. Thereafter, 100 μL of 0.25 mol/L pre-warmed calcium chloride was added and APTT was recorded as the time of clot formation. The TT assay was performed as follows: 90 μL of citrated normal human plasma was mixed with 10 μL of polysaccharide solution (0–50 μg/mL) and incubated at 37 °C for 60 s. Then, 200 μL of pre-warmed TT assay reagent (37 °C) was added to the mixture, and the clotting time was recorded. For PT clotting assay, 90 μL of citrated normal human plasma was mixed with 10 μL of polysaccharide solution (0–50 μg/mL) and incubated at 37 °C for 1 min. Then, 200 μL of pre-incubated PT assay reagent (37 °C, 10 min) was added to the mixture, and the clotting time was recorded. The clotting assays were performed in triplicate, and results are expressed as mean values ± standard deviations (SD) [15].

The effects on RAM264.7 cell proliferation and pinocytic activity assay in vitro was studied. The effect of polysaccharides on the viability of RAW264.7 cells was determined using the [3-(4,5-dimethylthiazol-2-yl)-2,5-diphenyltetrazolium] bromide (MTT) assay, which is based on the reduction of a tetrazolium salt by mitochondrial dehydrogenase in viable cells. After pre-incubating RAW264.7 cells (1 × 10^4^ cells/mL) for 18 h, polysaccharides (0–500 μg/mL) or LPS(10 μg/mL) was added and the mixture was incubated for an additional 24 h. 50 μL of the MTT stock solution (2 mg/mL) was then added to each well to attain a total reaction volume of 200 μL. After incubation for 2 h, the plate was centrifuged at 800 g for 5 min and the supernatants were aspirated. The formazan crystals in each well were dissolved in 150 μL dimethylsulfoxide and the A570 was read on a scanning multiwell spectrophotometer (JianCheng Bioengineering, Nanjing, China).

RAW264.7 cells were seeded at 1 × 10^4^ cells/well in a 96-well plate and incubated at 37 °C in a humidified atmosphere with 5% CO_2_. After 24 h, DMEM medium, LPS, or the various concentrations of polysaccharides (50, 100, 200, 400 μg/mL) were added into each well, and these cells were incubated at 37 °C for 24 h. Each concentration was repeated in three wells. Culture media were removed, and 100 μL /well of 0.075% neutral red was added and incubated for 30 min. After washed with PBS three times, 150 μL of cell lyzing solution were added into each well, and cells were cultured at 37 °C for 1 h. The absorbance was evaluated in an ELISA reader at 570 nm. Pinocytic ability (%) = (Asample/Acontrol − 1) × 100, where Acontrol was the absorbance of control without the tested samples, and Asample is the absorbance in the presence of the tested samples [24].

The data were expressed as mean ± standard deviation (S.D.) and examined for their statistical significance of difference with ANOVA. P-values of less than 0.05 were considered to be statistically significant.

## 4. Conclusions

In this paper, a sulfated low molecular weight polysaccharide, designated UP2-1, was isolated successfully from the marine green algae *Ulothrix flacca*. Chemical and structural characteristics analysis strongly suggested that UP2-1 is glucuronorhamnan, a typical ulvan in green algae. Bioactivity results demonstrated that UP2-1 had significant anticoagulant activity by prolonging APTT and TT. Although this activity was weaker than that of heparin, UP2-1 may be considered a potential anticoagulant agent. UP2-1 was also shown in this work to have immunomodulatory properties. Further investigations are needed to clarify, with greater detail, the anticoagulant effects and immunoregulation mechanisms of UP2-1. The marine green algae *Ulothrix flacca* is worth evaluating further as a candidate for functional food supplements or compounds suitable for the pharmaceutical industry. *U. flacca* is used as a traditional marine herb. It reminds us that polysaccharide might be an important active ingredient in this seaweed. We can also get inspiration from its traditional application and carry out a targeted bioactive study by modern technology to promote the development of traditional herbs.

## Figures and Tables

**Table 1:** GC–MS data analysis of the partial *O*-methylated alditol acetates of UP2-1 and dsUP2-1.

Retention Time (min)	Methylation Product	Molar Ratio (%)		Linkage Pattern
		UP2-1	dsUP2-1	
27.40	1,4,5-Tri-*O*-acetyl-2,4-di-*O*-metyl-L-Rha	9	67	→4)Rha(1→
27.50	1,3,5-Tri-*O*-acetyl-2,4-di-*O*-metyl-L-Rha	10	15	→3)Rha(1→
29.35	1,3,4,5-Tetr*o-O*-acetyl-2-*O*-metyl-L-Rha	70	8	→3,4)Rha(1→
30.15	1,2,4,5-Tetro-*O*-acetyl-3-*O*-metyl-L-Rha	11	10	→2,4)Rha(1→

**Table 2:** ^1^H and ^13^C NMR chemical shift assignment for the main sugar units of UP2-1.

Residues	C1/H1	C2/H2	C3/H3	C4/H4	C5/H5	C6/H6
A	102.3	70.9	71.0	81.2	71.3	18.5
(1→4)-α-L-Rha	5.01	4.01	4.10	4.00	3.78	1.32
B	100.1	70.0	79.6	76.4	71.1	18.5
(1→4)-α-L-Rha3S	4.93	4.23	4.61	3.52	4.08	1.32
C	104.4	3.3	3.55	3.64	-	-
(1→4)-β-D-GlcA	4.64	73.4	75.7	77.6	-	176

**Table 3:** Anticoagulant effects of UP2-1 on activated partial thromboplastin time (APTT), thrombin time (TT), and prothrombin time (PT) compared with heparin.

Index	Concentration (μg/mL)	0	2.5	5	10	20	50
APTT (s)	UP2-1	36.2 ± 4.5	48.8 ± 4.1	80.5 ± 1.9	130.4 ± 4.5	>200	>200
	Heparin	36.2 ± 4.5	90.5 ± 4.2	115.8 ± 3.7	>200	>200	>200
TT (s)	UP2-1	17.9 ± 3.1	24.5 ± 3.6	51.6 ± 2.7	118.8 ± 2.4	>120	>120
	Heparin	17.9 ± 3.1	68.8 ± 3.8	>120	>120	>120	>120
PT (s)	UP2-1	13.0 ± 2.3	15.5 ± 2.1	15.8 ± 1.6	16.8 ± 2.5	17.7 ± 2.9	19.6 ± 1.7
	Heparin	>120	47.6 ± 2.2	58.1 ± 3.2	67.2 ± 2.8	86.9 ± 3.3	>120

Each value is expressed as mean ± SD (n = 3).
